# Safety of a topical insect repellent (picaridin) during community mass use for malaria control in rural Cambodia

**DOI:** 10.1371/journal.pone.0172566

**Published:** 2017-03-24

**Authors:** Somony Heng, Vincent Sluydts, Lies Durnez, Vanna Mean, Koh Polo, Sochantha Tho, Marc Coosemans, Johan van Griensven

**Affiliations:** 1 National Center for Parasitology, Entomology and Malaria Control, Phnom Penh, Cambodia; 2 Institute of Tropical Medicine, Antwerp, Belgium; 3 University of Antwerp, Department of Biology, Antwerp, Belgium; 4 Banlong Referral Hospital, Ban Lung City, Ratanakiri Province, Cambodia; 5 University of Antwerp, Department of Biomedical Sciences, Antwerp, Belgium; Universite de Limoges, FRANCE

## Abstract

**Background:**

While community distribution of topical repellents has been proposed as an additional malaria control intervention, the safety of this intervention at the population level remains poorly evaluated. We describe the safety of mass distribution of the picaridin repellent during a cluster-randomised trial in rural Cambodia in 2012–2013.

**Methods:**

The repellent was distributed among 57 intervention villages with around 25,000 inhabitants by a team of village distributors. Information on individual adverse events, reported by phone by the village distributors, was obtained through home visits. Information on perceived side effects, reported at the family level, was obtained during two-weekly bottle exchange. Adverse events were classified as adverse reactions (events likely linked to the repellent), cases of repellent abuse and events not related to the repellent use, and classified as per Common Terminology Criteria for Adverse Events.

**Findings:**

Of the 41 adverse events notified by phone by the village distributors, there were 22 adverse reactions, 11 cases of repellent abuse (6 accidental, 5 suicide attempts) and 8 non-related events. All adverse reactions were mild, occurred in the first few months of use, and mainly manifested as skin conditions. Of the 11 cases of abuse, 2 were moderate and 2 life-threatening. All cases with adverse reactions and repellent abuse recovered completely. 20% of families reported perceived side effects, mainly itching, headache, dizziness and bad smell, but few discontinued repellent use.

**Conclusions:**

Adverse reactions and abuse during mass use of picaridin were uncommon and generally mild, supporting the safety of the picaridin repellent for malaria control.

## Introduction

Around 55 of the 106 countries with ongoing malaria transmission in 2000 are still on track to meet the World Health assembly target of a 75% reduction in malaria incidence by 2015 [1]. Effective vector control via large-scale use of long lasting insecticidal nets (LLINs) or indoor residual spraying (IRS) in combination with prompt and effective management of malaria cases, contributed largely to the malaria decline worldwide. However, residual malaria transmission caused by early evening and outdoor biting vectors is not covered by present vector control interventions [2, 3]. Therefore, additional vector control tools such as topical repellents need to be evaluated to further advance malaria elimination [4, 5].

A large-scale cluster randomized trial (MalaResT project) was set-up to evaluate the epidemiological impact of topical repellents in addition to the existing vector control measures (LLIN only) in the province of Ratanakiri in Cambodia [6]. To achieve community protection, mass distribution and use of repellents was promoted in the intervention arm aiming to reduce the residual malaria transmission. For over 50 years, DEET has been the most popular insect repellent worldwide. More recently, a synthetic component, namely picaridin, has been developed and is now also endorsed by the World Health Organization (WHO) [7]. In this study, picaridin was chosen as the repellent for its safety profile and excellent efficacy [7, 8]. Moreover, compared to DEET,[9, 10] it has a higher acceptability in terms of odor perception and does not affect plastic materials [11, 12].

Besides a randomized trial showing no adverse neurologic, gastrointestinal, or dermatologic effects among pregnant women applying daily DEET and no adverse effects on survival, growth, or development at birth or at one year among their babies [13], safety information of commonly used repellents mainly comes from studies conducted in small groups of healthy volunteers from high income countries when used for personal protection [11, 14]. We found no single study providing detailed safety information of repellent use when this is massively introduced in the community as part of a public health intervention. In contrast with earlier studies whereby short term exposure was assessed, the use of a topical repellent as a malaria control intervention implies daily skin application during an entire malaria season. Importantly, the tolerance and safety of the employed repellents might critically determine adherence to their use and hence also the effectiveness of the intervention. The aim of this study was to report on safety issues during the mass introduction of the picaridin repellent. Specifically, we report on the frequency of adverse reactions (ARs) reported individually in an expedited manner, and of perceived side effects reported per family during the two-weekly bottle exchanges.

## Materials and methods

### Study area and population

This study was part of a cluster randomized trial (MalaResT project), conducted in 113 hilly and mountainous villages in Ratanakiri province, eastern part of Cambodia, from January 2012 to December 2013. In 2012, the province had a population of 180,570 inhabitants (mainly subsistence farmers) spread over 240 main villages. The province contains 11 health centers, 19 health posts and a provincial referral hospital. The study area covered nine health centers; the provincial referral hospital was involved in the management of the AR and cases of repellent abuse.

### Study design

**Fig 1:**
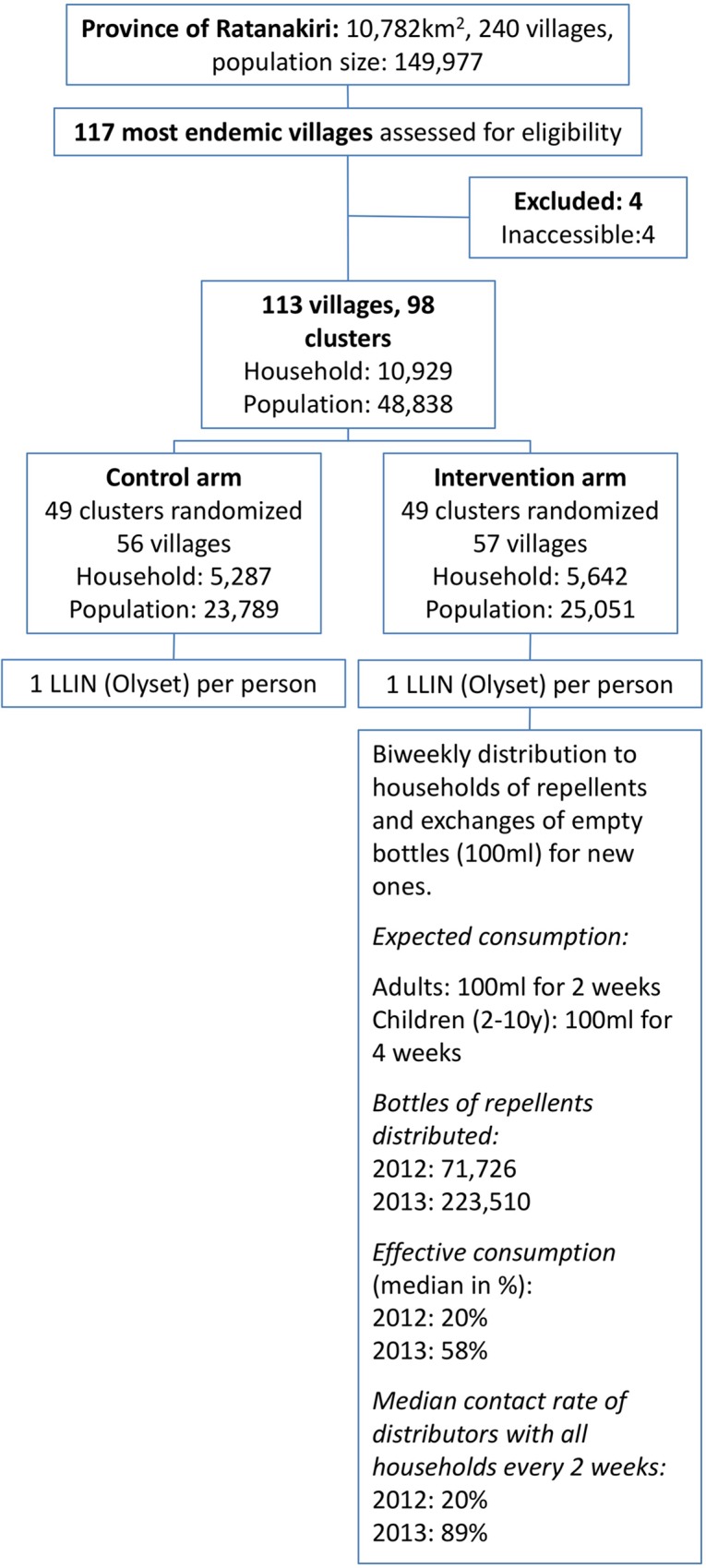
Flow chart describing the cluster randomized trial, evaluating the epidemiological efficacy of the introduction of insect repellents at the village level on malaria prevalence. Safety data reported in the present study come from the intervention arm.

The randomized cluster trial was composed of two arms, with clusters consisting of villages, conducted over two years. In the control arm (49 clusters; 56 villages; 5,287 families; 23,789 inhabitants) all individuals were provided with a LLIN (Fig 1).

In the intervention arm (49 clusters; 57 villages; 5,642 families; 25,051 inhabitants), besides the LLIN, all people aged over 2 years were additionally provided with the picaridin repellent to use during the early evening and morning when not protected by their bed nets. Children from 2 to 10 years were given picaridin 10% (as a milky lotion); all individuals above 10 years received picaridin 20% (in spray formulation). Both formulations applied at a dosage of 0.5ml/1000cm^2^ were shown to provide an effective protection during at least five hours [15]. No placebo repellent was used in the control arm as its use would have created a false perception of protection.

### Picaridin repellent, distribution & promotion

A chemical analysis of both formulations by an independent laboratory (Walloon Agriculture Centre, Belgium, a WHO collaborative center) showed agreement with the WHO specification [16] for the active ingredient content and the detection of impurities (sec-butyl chloroformate, sec-butyl carbonic anhydride) [17] (see S1 Text and S2 Text)

**Fig 2:**
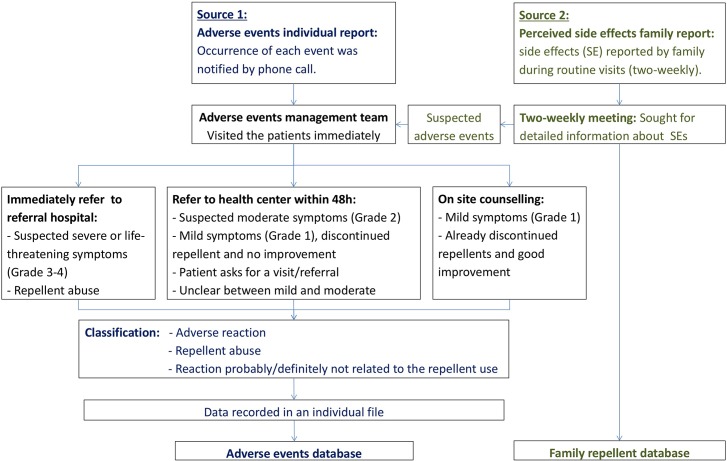
Information flow on AEs, perceived side effects and patient management tree.

The distribution of picaridin was done from April to December in 2012 and from March to December in 2013, covering the period of malaria transmission. A 100 ml bottle of picaridin (spray or lotion) is sufficient to cover the consumption of an adult and a child for two and four weeks respectively. A two-weekly bottle exchange program, performed by 135 village distributors, was put in place to ensure continuous access to the repellent. Distributors were supervised by nine supervisors from the health centers. In 2013, besides the health center supervisors, 13 additional supervisors were recruited to improve the repellent distribution, promote its use and facilitate data collection. All field activities were under the management of a field coordinator (who is a medical doctor) with two assistants, and all the AEs were given medical care by the field coordinator and another medical doctor based in the Ratanakiri provincial referral hospital (Fig 2).

The actual monthly repellent consumption was calculated per family expressed as a percentage of the expected consumption in 2012–2013. Repellent distribution started in 2012 in April, was interrupted during the dry season (January- February) and starting again in March 2013.

Every two weeks, all distributors met their respective supervisor at the health center to report on the activities and replenish the stock for the subsequent bottle exchange round [18]. During the entire study period, all users and care takers (for children) were informed about the importance and the appropriate use of the repellent, and how to react in case of AEs.

To promote compliance, a series of health education campaigns were conducted each year. In 2013, more intensive and continuous campaigns were performed.

### Data collection

Two types of information were used: 1) AE reported individually by phone and 2) Perceived side effects reported per family to the distributors during routine visits (Fig 2).

### AEs reported individually

Village health workers and distributors were trained to inform the field coordinator immediately by phone for any suspected AEs regardless of the causal relationship and all cases of picaridin abuse (intentional or accidental), with or without symptoms. All AEs and cases of repellent abuse were considered as urgent and followed by a visit of a study physician within 24 hours.

All information on such cases was recorded by the study physician on a specific template, reported to the trial sponsor within 24 hours and entered in a MS Access database (Adverse Events Database). All cases were promptly treated free of charge by the project medical doctors or referred to Ratanakiri provincial referral hospital. AEs were classified according to the protocol for report, management and treatment of AEs of the MalaResT (Fig 2), adapted from the “Common Terminology Criteria for AEs (CTCAE), version 4.0 [19] (see Box 1 for key definitions). For each reported AE, the types of expected and unexpected events were classified as per CTCAE criteria, the severity and likely causality with picaridin was determined.

**Box 1. Definitions used for AEs**

- *Adverse event*: is defined, for the scope of this study, as any untoward, undesired, or unplanned event in the form of signs, symptoms, disease, physiologic observations, or abuse (regardless of symptoms) occurring in a human being in a temporal relationship to the use of picaridin, regardless of causal relationship, including any clinically important worsening of a pre-existing condition.
- *Abuse*: any events that occurred due to the use of repellent besides skin application was called *abuse*, e.g. oral ingestion. This could be accidental (*e*.*g*. swallowing the product by a child) or intentional (e.g. suicide attempt).
- *Adverse reactions*: are AEs that are possibly, probably or definitely related to the use of the picaridin.
- *Causality*:
  ○ *Definitely related*: the event can be fully explained by administration of picaridin and/or a re-challenge was positive.
  ○ *Probably related*: the event is more likely to be explained by administration of picaridin rather than the patient/subject’s clinical state or other agents/therapies.
  ○ *Possibly related*: the event may be explained by administration of picaridin, or by the patient/subject's clinical state or other agents/therapies.
  ○ *Probably not related*: the event is more likely to be explained by the patient/subject's clinical state or other agents/therapies rather than picaridin.
  ○ *Definitely not related*: the event can be fully explained by the patient/subject's clinical state or other agents/therapies.
- *Severity*:*—Grade 1*: mild.—*Grade 2*: moderate.—*Grade 3*: severe.—*Grade 4*: Life-threatening consequences
- *Serious adverse events*: life threatening AE that requires inpatient hospitalization, results in a persistent or significant disability or incapacity, in cancer, in a congenital anomaly or birth defect or the patient is required medical or surgical intervention to prevent unwanted outcomes.
- *Expected adverse reaction*: mild to moderate allergic contact dermatitis and eye irritation.
- *Unexpected adverse reaction*: any other mild to moderate reactions as mentioned above and any severe ARs.
- Case definition according to CTCAE [19]:
  ○ *Dyspnea*: *uncomfortable* sensation of difficulty breathing.
  ○ *Dry skin*: flaky *and* dull skin, the pores are generally fine, the texture is a papery thin texture.
  ○ *Maculo-papular rash*: the *presence* of macules (flat) and papules (elevated). Also known as morbilliform rash (rash resembling that of measles), it is one of the most common cutaneous AEs, frequently affecting the upper trunk, spreading centripetally and associated with pruritus.
  ○ *Papulopustular rash*: an eruption consisting of papules (a small, raised pimple) and pustules (a small pus filled blister), typically appearing in face, scalp, and upper chest and back. Unlike acne, this rash does not present with whiteheads or blackheads, and can be symptomatic, with itchy or tender lesions.
  ○ *Pruritus*: intense itching sensation.
  ○ *Pain of skin*: marked discomfort sensation in the skin.
  ○ *Un-identified*: any events that did not match these CTCAE definitions.

### Perceived side effects reported per family to the distributors during routine visits

During each bottle exchange round, the distributors interviewed all family representatives and recorded information regarding: the amount of repellent remaining in each bottle, the perceived side effects (using open-ended questions, see Box 2) by any family member and the reasons for not using the repellent. All information was recorded in a household data sheet per family and per bottle exchange (S1 Table), and entered in an MS Access database (Family Repellent Database). All sheets were checked for completeness, and all reported complaints were discussed during the two-weekly meetings. Serious complaints were treated as suspected AEs and handled accordingly (Fig 2).

**Box 2 Interpretation and grouping of perceived side effects reported per family to the distributors during routine visits. (The definitions below are the literal English translations of how these concepts are expressed in the local language)**

- *Perceived side effects*: side effects occurring during the two weeks among the family members and reported by the family representative (open question: see S3 Table).
- *Bad smell*: user’s complaint about unpleasant smell such as: “not good, bad smell, or strong smell”.
- *Dizziness*: feeling that things around her/him turning around and s/he is not able to keep her/his balance, possibly leading to falling down.
- *Headache*: “feeling pain inside the head” or “heavy head”.
- *Itching*: unpleasant feeling on the skin making her/him want to scratch.
- *Irritation*: feeling pain (and hot) on the skin.
- *Rash*: appearance of small red spots on the skin.
- *Nausea*: feeling a tendency to vomit, but without actually vomiting.
- *Vomiting*: unwillingly emptying the contents of the stomach through the mouth.

### Data analysis

#### AEs individually reported

The data were categorized as ARs or cases of repellent abuse. AEs were classified as AR when the medical doctor who visited the patient judged a possible, probable or definite causal relation with the repellent use was in place. An AE was classified as repellent abuse if the topical repellent was orally ingested (Box 1). The median age and percentage of cases by sex, case definition, preexisting status, causality, severity, repellent use continuation, treatment received and improvement status were reported for each AR and case of repellent abuse. We also calculated the annual incidence rate per 1000 inhabitants of ARs and cases of repellent abuse. Cases of reactions probably or definitely not related to the repellent use were excluded from the analysis.

#### Perceived side effects reported per family to the distributors during routine visits

The percentage of families reporting perceived side effects among families receiving picaridin, and when this occurred after starting the repellent use, was calculated for 2012 and 2013 separately. A family could report the same perceived side effect several times at different bottle exchanges. A family that had reported any perceived side effect, regardless of frequency, was considered as “having reported perceived side effects”. The average of the actual monthly repellent consumption per family was expressed as the percentage of the expected monthly consumption. This was calculated by dividing the average amount of repellent (ml) all families actually received per month by the expected monthly consumption per family. The expected monthly consumption for a child is 100 ml and 200 ml for an adult.[18] All analysis was done in R version 3.1.1. The study was conducted following STROBE guidelines (see S3 Text).

#### Ethical considerations

This study was part of the MalaResT project which was registered as NCT01663831. At the beginning of each year, a written community consent form was given to each village chief to get his/her approval to conduct the study in his/her village. All villagers were invited to participate on a voluntarily basis in the study. Since a vast majority of the villagers were unable to write or read, they were verbally informed by distributors in local languages during the two-weekly bottle exchange about the objectives of the repellent distribution and data collection. Those who agreed to participate were given exchange bottles and asked to sign or stamp a finger print on “*Distribution of repellent bottles and recovery of empty bottles*” form (S1 Table), otherwise this form was not documented. The study protocol (see S4 Text) including this consent procedure was approved by the Institutional Review Board of the Institute of Tropical Medicine, Antwerp (Approval IRB/AB/ac/154), the Ethics Committee of the University of Antwerp (Approval B300201112714), Belgium and the Cambodian National Ethics Committee for Health Research of Ministry of Health (Approval 265 NECHR).

#### Role of funding source

The funders of the study had no role in study design, data collection, data analysis, data interpretation, or writing of the report. The corresponding author had full access to all the data in the study and had final responsibility for the decision to submit for publication.

## Results

### AEs individually reported

Over the two years period a total of 41 individual AEs were individually reported by phone. 22 were classified as ARs and 11 as repellent abuse (Table 1 and Table 2). The remaining eight were judged not related to the repellent (details in S2 Table). All ARs were considered mild. Of the cases of repellent abuse, three were considered moderate and two as life-threatening.

**Table 1:** ARs notified by phone and their classification according to the terminology of the study protocol for reporting, management and treatment of AEs (N = 22).

Variable		N	%
**Sex:**	Female	14	63.6
	Male	8	36.4
**Median age—years** (interquartile range)		29 (17–60)	
**Cutaneous application**		22	100.0
**Expected adverse reaction** (according to CTCAE classification):		21	95.5
	Pruritus	11	50.0
	Papulo-pustular rash	7	31.8
	Maculo-papular rash	2	9.1
	Dry skin	1	4.5
**Un-expected AE** (according to CTCAE classification):		1	4.5
	Un-identified**	1	4.5
**Pre-existing:**			
	Yes, but did not get worse	2	9.1
	Yes, did get worse	1	4.5
	No	19	86.4
**Relationship to study treatment (causality):**			
	Definitely related	20	90.9
	Probably related	1	4.5
	Possibly related	1	4.5
**Mild severity:**		22	100.0
**Serious adverse event:**		0	0
**Further repellent use:**			
	No discontinuation	4	18.2
	Permanently discontinued	17	77.3
	Temporarily discontinued	1	4.5
**Treatment:**			
	No	14	63.6
	Topical steroid and/or antihistamine	5	22.7
	Washing	3	13.6
**Improved:**		22	100

*CTCAE*: Common Terminology Classification for Adverse Event. All cases occurred in 2012.

** Unidentified case: A 60 years old man having bitter taste in the mouth and weakness after applying the repellent.

**Table 2:** Repellent abuse (oral ingestion) notified by phone and their classification according to the terminology of the study protocol for reporting, management and treatment of AEs (N = 11).

Variable		n	%
**Year of occurrence:**			
	2012	5	45.5
	2013	6	54.5
**Sex**			
	Female	5	45.5
	Male	6	54.5
**Median age—years** (interquartile range):		19 (6–25)	
**Type of use:**			
	Accidental	6	54.5
	Suicide attempt	5	45.5
**Relationship to study treatment:**			
	Definitely related	11	100.0
**Un-expected:**		11	100.0
**Severity:**			
	Mild	5	45.5
	Moderate	3	27.3
	Life-threatening	2	18.2
	Asymptomatic	1	9.1
**Further repellent use:**			
	Continued use	8	72.7
	Temporarily discontinued use	3	27.3
**Hospitalized:**			
	Yes	3	27.3
	No	8	72.7
**Improved:**		11	100.0

**Fig 3:**
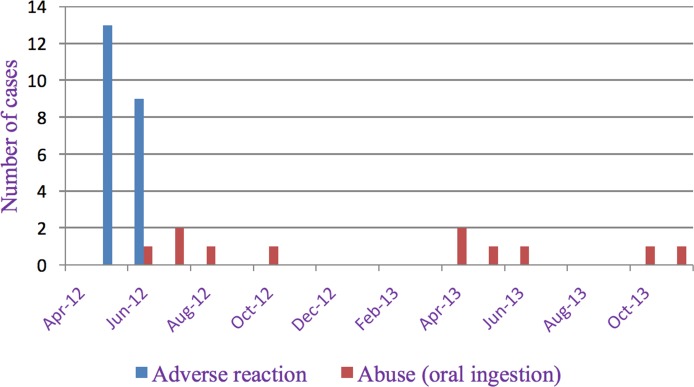
Monthly counts of ARs and repellent abuses (oral ingestion), 2012–2013.

All 22 ARs occurred in six villages in 2012 in different families belonging to six villages (Fig 3).

Most cases were female having skin manifestations (see details in Table 1 and S2 Table). Three cases were children under or equal to ten years old (S2 Table). In fourteen cases, the event occurred during the first two weeks of application. Seventeen cases (77.3%) were advised by the study physician to permanently stop using the repellent. All cases completely recovered, of which 14 (63.6%) without treatment (Table 1). The total incidence rate per 1,000 inhabitants of ARs for all intervention villages was 0.84 in 2012 but ranged from 1.05 to 17.63 in the affected 6 villages (Table 3).

**Table 3:** Incidence rate per 1000 inhabitants of ARs notified by phone, 2012.

Village code	Population	Adverse reaction	Incidence rate/1000 inhabitants
3378	952	1	1.05
3331	840	2	2.38
3260	323	3	9.29
3393	791	4	5.06
3255	326	5	15.34
3181	397	7	17.63
Total (6 villages)	3,629	22	6.06
Total (all 57 intervention villages)	26,216	22	0.84

No adverse reaction notified by phone in 2013.

There were five cases of repellent abuse in 2012 and six in 2013, involving nine villages (see details in Table 2, Fig 3 and S3 Table). On six occasions the oral ingestion was accidental, but on five it was considered a suicide attempt. All suicide attempts were women ranging from 13 to 26 years of age. Of the six accidents, four were male children between 2 and 7 years of age.

The mild and moderate cases typically presented with nausea, vomiting, headache and fatigue. One mild case involved a 4 months pregnant woman who attempted to commit suicide by drinking about 50 ml (a half bottle) of 20% picaridin. Fortunately, no negative impact on fetus and new born was noted. One moderate case consisted of convulsions in a 25-year old lady with a history of childhood epilepsy. The two life-threatening suicide attempt cases, in girls aged 19 and 13 years old, were unconscious for about two hours after drinking 200–300 ml (two to three full bottles). Besides unconsciousness, the two patients always had normal vital signs recorded in the health facilities. The treatment of the first life-threatening case included administration of intravenous fluids and atropine. The second life-threatening case was treated in the village with administration of intravenous fluids, cortico-steroids and antibiotics. After treatment, all cases completely recovered. The total incidence rate of repellent abuse per 1000 inhabitants was 0.2 in both years for all intervention villages, while the incidence for villages ranged from 1.9 to 3.5 and 1 to 5.7 in 2012 and 2013 respectively (Table 4).

**Table 4:** Incidence rate per 1000 inhabitants of repellent abuses, 2012–2013.

Village code	Population 2012	Population 2013	Abuse 2012	Abuse 2013	Incidence per 1000–2012	Incidence per 1000–2013
3331	840	839	2	0	2.4	0
3423	520	499	1	0	1.9	0
3204	578	576	2	0	3.5	0
3179	716	699	0	1	0	1.4
3393	791	568	0	1	0	1.8
3244	579	548	0	1	0	1.8
3378	952	1033	0	1	0	1.0
3326	167	175	0	1	0	5.7
3332	220	227	0	1	0	4.4
Total 9 villages	5363	5164	5	6	0.9	1.2
Total all 57 intervention villages	26,216	25121	5	6	0.2	0.2

### Perceived side effects reported by the families to the distributors during routine visits

**Fig 4:**
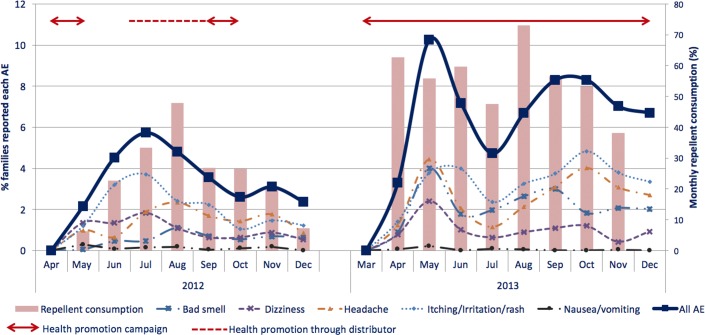
Monthly trends of families reporting perceived side effects and average of the actual monthly repellent consumption.

More than 95% of all families received the repellent at least once (Table 5). Around 20% of the families receiving picaridin reported perceived side effects each year, half of them occurring within the first two to three months of use (Table 5 & Fig 4).

**Table 5:** Families reported perceived side effects and repellent consumption through distributor reports, 2012–2013. A percentage above 100% of families receiving repellents in 2013 is due to new arrivals after the population census done early 2013 were given repellent and recorded but were not updated in the census data.

Indicator				2012 (n; %), N = 5809	2013 (n; %), N = 5642
Families receiving repellents:				5518; 95.0%	5852; 103.7%
	- Families reporting any perceived side effects:			1116; 20.2%	1231; 21.0%
		- Frequency of reporting any perceived side effects:			
			- 1	766; 68.6%	607; 49.3%
			- 2	251; 22.5%	196; 15.9%
			- > = 3	99; 8.9%	428; 34.8%
		- Time to occurrence of first perceived side effects(s) (day):		Median = 83; Q1 = 51 Q3 = 132	Median = 62; Q1 = 37 Q3 = 128
	- Average of the actual monthly repellent consumption for all families expressed as a percentage of the expected monthly consumption:				
			- All families	23.9% (SD = 12.6%)	60% (SD = 14.7%)
			- Families with adverse event(s)	7.3% (SD = 4.9%)	5.6% (SD = 1.9%)
			- Families without adverse event	28.8% (SD = 15.7%)	76.5% (SD = 18.8%)
	- Families reporting perceived side effects(s). One family can report one or more side effects:				
			- Headache	483; 8.8%	681; 11.6%
			- Bad smell	222; 4.0%	596; 10.2%
			- Itching/irritation/rash	696; 12.6%	589; 10.1%
			- Dizziness	417; 7.6%	321; 5.5%
			- Others (cold, cough, fever…)	118; 2.1%	97; 1.8%
			- Nausea/vomiting	54; 1.0%	25; 0.4%

Of those families, most (68.6%) reported side effects only once in 2012, while in 2013 about half reported two times or more. In both years, families reporting perceived side effects consumed less repellent. In 2012, itching/irritation/rash (12.6%), headache (8.8%) and dizziness (7.6%) were the top three reported side effects. In 2013, headache (11.6%), bad smell (10.2%) and itching/irritation/rash (10.1%) were most common (Table 5).

## Discussion

This study assessed the safety of the mass introduction of a topical insect repellent in rural communities as part of a public health malaria control intervention. An extensive reporting system was put in place to detect AEs associated with the use of the repellent, applied twice daily for an extended period of time. On a total of about 25,000 individual repellent users from the intervention arm, only 22 –less than one in thousand—ARs related to cutaneous repellent use and 11 cases of repellent abuse were reported.

The ARs related to topical use of the repellent occurred during the first few months after the introduction of the repellent in the population, and mostly during the first two weeks of use (Fig 3). Contact or irritant dermatitis is indeed to be expected within the first days or weeks of use, and individuals with such reactions generally discontinued repellent use. Inappropriate repellent use during the early stages of the project might have contributed to some of the ARs. As observed during field visits, some individuals continued, sometimes encouraged by their distributor to use the repellent despite skin reactions, and this apparently led to progressive worsening. Only 326 (5.6%) of families were recorded by the distributors to discontinue the use of the repellent during the trial.

Repellent abuse (either accidental or suicide attempt) is another concern for community level interventions. Children and particularly young boys were at risk for accidental ingestion of the product. All suicide attempts occurred in young women, which is in line with the fact that within the region, women are more likely to attempt suicide and to use less-violent methods [20, 21]. The insect repellent DEET has been associated with neurotoxic effects such as seizures, although causality was difficult to demonstrate [22–24]. As to picaridin, no neurotoxicity or lethal effect have been reported in animal experiments and individual human experience [8, 25]. Our findings demonstrate that hallucinations and unconsciousness can occur after ingestion of high amounts of picaridin (2–3 bottles, 200–300 ml), but reassuringly this resolved completely with supportive treatment. There was one case of intentional abuse in a pregnant woman, who delivered a healthy baby. In animal experiments, maternal effects such as increased liver weights were observed with application of picaridin, but no toxic effects on the fetuses was detected [25].

In 2013, there was an intensification of the health promotion campaigns, and concurrently more perceived side effects were reported. Similarly, within each year, periods with more repellent use appeared to be associated with more perceived side effects and families reporting side effects also had a lower average repellent consumption. We note however that the increased reporting of side effects during the second year was largely explained by increased reporting of headache, dizziness and bad smell, while the frequency of skin manifestations remained fairly stable across the two years. Importantly, there were no indications of cumulative toxicity in our study, whereby side effects would have started emerging only after prolonged use. This is reassuring for a malaria control intervention which implies daily administration possibly over a period of several years.

Bad smell associated with headache and dizziness, has been reported related to agrochemical products [26]. In a comparative study performed in Malaysia participants felt more comfortable with the odor of picaridin as compared to DEET [27]. As preferences in smell can vary extensively geographically and across societies, adapting the repellent’s perfume to local preferences might be a way to improve adherence.

Our findings need to be interpreted in the specific context of the study. First, the reported findings were observed during a cluster-randomized trial, whereby due efforts were done to carefully inform users about the appropriate use and what to do in case of AEs, and whereby users were closely monitored for intolerance. We also note that we did not collect information on AEs and perceived side effects in the control arm, since no placebo was used. This makes it obviously difficult to causally link the spontaneously reported symptoms to the repellent use, and our findings are thus most likely an overestimation. Finally, the aggregation at the family level of data on perceived side effects precluded more detailed individual level analysis.

In conclusion, we found ARs during mass introduction of the picaridin insect repellent to be uncommon and generally mild. All the 22 ARs reported, amongst over 25,000 individual users occurred during the first few months of the study. Oral repellent abuse was also uncommon but occurred throughout the two years. Hence, control programs employing mass repellent use should be particularly prepared to detect and manage ARs at the time of program onset and cases of repellent abuse remain throughout. While one in five families reported any side effect, few families discontinued repellent use. Our study supports the safety of the picaridin insect repellent for community wide use as part of a malaria control intervention.

## Supporting information

S1 Text CERTIFICATE OF ANALYSIS ITM / FO 23005 / Ch.5362 to 5365 / 2012 / A. (PDF)

S2 Text CERTIFICATE OF ANALYSIS ITM / FO 23458 / Ch.5774 / 2013 / A. (PDF)

S3 Text STROBE checklist. (DOC)

S4 Text Protocol for Reporting, Management and Treatment of Adverse Reactions topical repellents. (PDF)

S1 Table Household data sheet. (DOCX)

S2 Table Summary of adverse events individually reported. (DOCX)

S3 Table Summary of cases of repellent abuse (oral ingestion). (DOCX)
