# Supplementary material for: Safety of a topical insect repellent (picaridin) during community mass use for malaria control in rural Cambodia
Source: PLoS One. 2017 Mar 24;12(3):e0172566. doi: 10.1371/journal.pone.0172566 (PMC5365103; doi:10.1371/journal.pone.0172566)
Supplement: S1 Text — (PDF) [file pone.0172566.s001.pdf]

Walloon Agricultural Research Centre (CRA-W)
Agriculture and Natural Environment Department (D3)

cra-w

Wallonie

Plant Protection Products and Biocides
Physico-chemistry and Residues Unit (U10)
Carson Building
Rue du Bordia, 11
B - 5030 GEMBLOUX - Belgium
Phone : +32 (0) 81 62.52.62 - Fax : +32 (0) 81 62.52.72
E-mail : pesticides@cra.wallonie.be

B - 5030 - GEMBLOUX, December 19, 2012

Institute of Tropical Medicine Antwerp
Department of Biomedical Science
Unit of Medical Entomology
Nationalestraat 155
B-2000 Antwerpen
BELGIUM

To Prof. Marc Coosemans

CERTIFICATE OF ANALYSIS ITM / FO 23005 / Ch.5362 to 5365 / 2012 / A

Samples of : **Repellent spray**
Declared active substance content : icaridin 20% w/w
Manufacturer : SC Johnson
Batch number : MANF 170212 20 – A1 106018

Repellent spray
Declared active substance content : icaridin 20% w/w
Manufacturer : SC Johnson
Batch number : MANF 160212 03 – A1 005913

Repellent lotion
Declared active substance content : icaridin 10% w/w
Manufacturer : SC Johnson
Batch number : MANF 220212 70 – C1 074256

Repellent lotion
Declared active substance content : icaridin 10% w/w
Manufacturer : SC Johnson
Batch number : MANF 210212 76 – C1 039490

Supplier : Institute of Tropical Medicine Antwerp.

Arrived on : July 30, 2012.

Type of package : plastic flask of 100 mL.

Reference : Request of July 27, 2012 from Prof. Marc Coosemans (Institute of Tropical Medicine Antwerp).

RESULTS :

1. Icaridin content

[CIPAC method 740/TC/(M)/3, CIPAC Handbook K, p.65, Gas Chromatography with Flame Ionization Detection (GC-FID)].

Dates of analysis : from November 21 until November 22, 2012.

Repellent spray - Batch MANF 170212 20 – A1 106018

WHO general specification limit : 20% w/w ± 6% [18.8 – 21.2 % w/w]

Determination	Icaridin content (% w/w)
1	19.94
2	20.02
3	19.88
**Mean**	**19.95**
Standard deviation	0.07
Relative standard deviation (RSD) *	0.35 %
Confidence interval of the mean (Student T-test with a probability of 95 %)	**19.95 ± 0.17**

* RSD < RSD Horwitz x 0.67 (1.71 %).

Repellent spray - Batch MANF 160212 03 – A1 005913

WHO general specification limit : 20% w/w ± 6% [18.8 – 21.2 % w/w]

Determination	Icaridin content (% w/w)
1	19.95
2	20.19
3	19.67
**Mean**	**19.94**
Standard deviation	0.26
Relative standard deviation (RSD) *	1.30 %
Confidence interval of the mean (Student T-test with a probability of 95 %)	**19.94 ± 0.65**

* RSD < RSD Horwitz x 0.67 (1.71 %).

Repellent lotion - Batch MANF 220212 70 – C1 074256

WHO general specification limit : 10% w/w ± 10% [9.0 – 10.0 % w/w]

Determination	Icaridin content (% w/w)
1	9.08
2	9.20
3	9.07
**Mean**	**9.12**
Standard deviation	0.07
Relative standard deviation (RSD) *	0.77 %
Confidence interval of the mean (Student T-test with a probability of 95 %)	**9.12 ± 0.17**

* RSD < RSD Horwitz x 0.67 (1.92 %).

Repellent lotion - Batch MANF 210212 76 – C1 039490

WHO general specification limit : 10% w/w ± 10% [9.0 – 10.0 % w/w]

Determination	Icaridin content (% w/w)
1	9.75
2	9.77
3	9.78
**Mean**	**9.77**
Standard deviation	0.02
Relative standard deviation (RSD) *	0.20 %
Confidence interval of the mean (Student T-test with a probability of 95 %)	**9.77 ± 0.05**

* RSD < RSD Horwitz x 0.67 (1.90 %).

2. Impurity *sec*-butyl chlorformate content

[Method developed by the test facility and based on CIPAC method 740/TC/(M)/3, CIPAC Handbook K, p.65, and Gas Chromatography with Mass Spectrometry Detection (GC-MS)].

Dates of analysis : from November 21 until December 01, 2012.

Repellent spray - Batch MANF 170212 20 – A1 106018

Determination	***sec*-butyl chlorformate content**	
	**g/kg**	**g/kg of the icaridin content**
1	ND	ND
2	ND	ND
3	ND	ND
**Mean**	**ND**	**ND**
Standard deviation	-	-
Relative standard deviation (RSD)	-	-
Confidence interval of the mean (Student T-test with a probability of 95 %)	-	-

ND = not detected (limit of quantification = 0.005 g/kg or 0.025 g/kg of the icaridin content).

Repellent spray - Batch MANF 160212 03 – A1 005913

Determination	***sec*-butyl chlorformate content**	
	**g/kg**	**g/kg of the icaridin content**
1	ND	ND
2	ND	ND
3	ND	ND
**Mean**	**ND**	**ND**
Standard deviation	-	-
Relative standard deviation (RSD)	-	-
Confidence interval of the mean (Student T-test with a probability of 95 %)	-	-

ND = not detected (limit of quantification = 0.005 g/kg or 0.025 g/kg of the icaridin content).

Repellent lotion - Batch MANF 220212 70 – C1 074256

Determination	*sec*-butyl chlorformate content	
	**g/kg**	**g/kg of the icaridin content ****
1	0.008	0.084
2	0.008	0.086
3	0.008	0.087
**Mean**	**0.008**	**0.086**
Standard deviation	0.000	0.002
Relative standard deviation (RSD) *	0.00 %	1.78 %
Confidence interval of the mean (Student T-test with a probability of 95 %)	**0.008 ± 0.000**	**0.086 ± 0.001**

* RSD < RSD Horwitz x 0.67 (5.54 %).
** Using the result obtained for icaridin content.

Repellent lotion - Batch MANF 210212 76 – C1 039490

Determination	*sec*-butyl chlorformate content	
	**g/kg**	**g/kg of the icaridin content ****
1	0.007	0.070
2	0.007	0.072
3	0.007	0.073
**Mean**	**0.007**	**0.072**
Standard deviation	0.000	0.002
Relative standard deviation (RSD) *	0.00 %	2.13 %
Confidence interval of the mean (Student T-test with a probability of 95 %)	**0.007 ± 0.000**	**0.072 ± 0.004**

* RSD < RSD Horwitz x 0.67 (5.66 %).
** Using the result obtained for icaridin content.

3. Impurity *sec*-butyl carbonic anhydride content

[Method developed by the test facility and based on CIPAC method 740/TC/(M)/3, CIPAC Handbook K, p.65, and Gas Chromatography with Mass Spectrometry Detection (GC-MS)].

Dates of analysis : from November 21 until December 01, 2012.

Repellent spray - Batch MANF 170212 20 – A1 106018

Determination	*sec*-butyl carbonic anhydride content	
	g/kg	g/kg of the icaridin content **
1	0.180	0.904
2	0.182	0.915
3	0.187	0.937
**Mean**	**0.183**	**0.919**
Standard deviation	0.004	0.017
Relative standard deviation (RSD) *	1.97 %	1.83 %
Confidence interval of the mean (Student T-test with a probability of 95 %)	**0.183 ± 0.009**	**0.919 ± 0.042**

* RSD < RSD Horwitz x 0.67 (3.46 %).
** Using the result obtained for icaridin content.

Repellent spray - Batch MANF 160212 03 – A1 005913

Determination	*sec*-butyl carbonic anhydride content	
	g/kg	g/kg of the icaridin content **
1	0.184	0.921
2	0.182	0.912
3	0.177	0.890
**Mean**	**0.181**	**0.908**
Standard deviation	0.004	0.016
Relative standard deviation (RSD) *	1.99 %	1.76 %
Confidence interval of the mean (Student T-test with a probability of 95 %)	**0.181 ± 0.009**	**0.908 ± 0.040**

* RSD < RSD Horwitz x 0.67 (3.47 %).
** Using the result obtained for icaridin content.

Repellent lotion - Batch MANF 220212 70 – C1 074256

Determination	*sec*-butyl carbonic anhydride content	
	g/kg	g/kg of the icaridin content **
1	0.092	1.008
2	0.096	1.055
3	0.092	1.005
**Mean**	**0.093**	**1.023**
Standard deviation	0.002	0.028
Relative standard deviation (RSD) *	2.47 %	2.74 %
Confidence interval of the mean (Student T-test with a probability of 95 %)	**0.093 ± 0.006**	**1.023 ± 0.070**

* RSD < RSD Horwitz x 0.67 (3.83 %).
** Using the result obtained for icaridin content.

Repellent lotion - Batch MANF 210212 76 – C1 039490

Determination	*sec*-butyl carbonic anhydride content	
	g/kg	g/kg of the icaridin content **
1	0.099	1.016
2	0.103	1.058
3	0.099	1.009
**Mean**	**0.100**	**1.028**
Standard deviation	0.002	0.027
Relative standard deviation (RSD) *	2.30 %	2.58 %
Confidence interval of the mean (Student T-test with a probability of 95 %)	**0.100 ± 0.006**	**1.028 ± 0.066**

* RSD < RSD Horwitz x 0.67 (3.79 %).
** Using the result obtained for icaridin content.

Dr. ir. Olivier PIGEON
Scientific Unit Coordinator
