# Supplementary material for: Safety of a topical insect repellent (picaridin) during community mass use for malaria control in rural Cambodia
Source: PLoS One. 2017 Mar 24;12(3):e0172566. doi: 10.1371/journal.pone.0172566 (PMC5365103; doi:10.1371/journal.pone.0172566)
Supplement: S2 Text — (PDF) [file pone.0172566.s002.pdf]

Walloon Agricultural Research Centre (CRA-W)
Agriculture and Natural Environment Department (D3)

cra-w   Wallonie

Plant Protection Products and Biocides
Physico-chemistry and Residues Unit (U10)
Carson Building
Rue du Bordia, 11
B - 5030 GEMBLOUX - Belgium
Phone : +32 (0) 81 62.52.62 - Fax : +32 (0) 81 62.52.72
E-mail : pesticides@cra.wallonie.be

B - 5030 - GEMBLOUX, December 10, 2013

Institute of Tropical Medicine Antwerp
Department of Biomedical Science
Unit of Medical Entomology
Nationalestraat 155
B-2000 Antwerpen
BELGIUM

To Prof. Marc Coosemans

CERTIFICATE OF ANALYSIS ITM / FO 23458 / Ch.5774 / 2013 / A

Samples of : **Repellent spray**
Declared active substance content : icaridin 20% w/w
Manufacturer : SC Johnson
Batch number : MANF 250113 65 - A2 094478

Supplier : Institute of Tropical Medicine Antwerp.

Arrived on : October 14, 2013.

Type of package : plastic flask of 100 mL.

Reference : Request of October 01, 2013 from Prof. Marc Coosemans (Institute of Tropical Medicine Antwerp).

RESULTS :

1. Icaridin content

[CIPAC method 740/TC/(M)/3, CIPAC Handbook K, p. 65, Gas Chromatography with Flame Ionization Detection (GC-FID)].

Dates of analysis : November 20, 2013.

Repellent spray - Batch MANF 250113 65 - A2 094478

WHO general specification limit : 20% w/w ± 6% [18.8 – 21.2 % w/w]

Determination	Icaridin content (% w/w)
1	20.02
2	20.02
3	19.98
**Mean**	**20.01**
Standard deviation	0.02
Relative standard deviation (RSD) *	0.10 %
Confidence interval of the mean (Student T-test with a probability of 95 %)	**20.01 ± 0.05**

* RSD < RSD Horwitz x 0.67 (1.71 %).

2. Impurity *sec*-butyl chlorformate content

[Method developed by the test facility and based on CIPAC method 740/TC/(M)/3, CIPAC Handbook K, p. 65, and Gas Chromatography with Mass Spectrometry Detection (GC-MS)].

Dates of analysis : from December 02 until December 03, 2013.

Repellent spray - Batch MANF 250113 65 - A2 094478

Determination	*sec*-butyl chlorformate content	
	g/kg	g/kg of the icaridin content
1	ND	ND
2	ND	ND
3	ND	ND
**Mean**	**ND**	**ND**
Standard deviation	-	-
Relative standard deviation (RSD)	-	-
Confidence interval of the mean (Student T-test with a probability of 95 %)	-	-

ND = not detected (limit of quantification = 0.005 g/kg or 0.025 g/kg of the icaridin content).

3. Impurity *sec*-butyl carbonic anhydride content

[Method developed by the test facility and based on CIPAC method 740/TC/(M)/3, CIPAC Handbook K, p. 65, and Gas Chromatography with Mass Spectrometry Detection (GC-MS)].

Dates of analysis : from December 02 until December 03, 2013.

Repellent spray - Batch MANF 250113 65 - A2 094478

Determination	*sec*-butyl carbonic anhydride content	
	g/kg	g/kg of the icaridin content **
1	0.173	0.864
2	0.175	0.873
3	0.179	0.894
**Mean**	**0.176**	**0.877**
Standard deviation	0.003	0.015
Relative standard deviation (RSD) *	1.74 %	1.76 %
Confidence interval of the mean (Student T-test with a probability of 95 %)	**0.176 ± 0.008**	**0.877 ± 0.038**

* RSD < RSD Horwitz x 0.67 (3.48 %).
** Using the result obtained for icaridin content.

December 10, 2013

Dr ir Olivier PIGEON
Scientific Unit Coordinator

cra-w
