# Supplementary material for: Safety of a topical insect repellent (picaridin) during community mass use for malaria control in rural Cambodia
Source: PLoS One. 2017 Mar 24;12(3):e0172566. doi: 10.1371/journal.pone.0172566 (PMC5365103; doi:10.1371/journal.pone.0172566)
Supplement: S3 Text — (DOC) [file pone.0172566.s003.doc]

STROBE Statement—Checklist of items that should be included in reports of ***cohort studies***

|  | Item No | Recommendation |
| --- | --- | --- |
| **Title and abstract** | 1 | (*a*) Indicate the study’s design with a commonly used term in the title or the abstract  **Design explained in abstract (Background/methods)** |
|  |  | (*b*) Provide in the abstract an informative and balanced summary of what was done and what was found  **Covered in the different sections of the structured abstract** |
| Introduction | | |
| Background/rationale | 2 | Explain the scientific background and rationale for the investigation being reported  **Covered in Background, especially the last paragraph (page 3)** |
| Objectives | 3 | State specific objectives, including any prespecified hypotheses  **Provided in the last two sentence of the Background page 3)** |
| Methods | | |
| Study design | 4 | Present key elements of study design early in the paper  **Elements of study design introduced in Background (page 3) and further developed in section 2 of the Methods (page 4: study design).** |
| Setting | 5 | Describe the setting, locations, and relevant dates, including periods of recruitment, exposure, follow-up, and data collection.  **Provided in the Methods ( page 4,5).** |
| Participants | 6 | (*a*) Give the eligibility criteria, and the sources and methods of selection of participants. Describe methods of follow-up  **Provided in Methods section (study population, study design, Picaridin repellent, distribution & promotion – page 4, 5)** |
|  |  | (*b*) For matched studies, give matching criteria and number of exposed and unexposed |
| Variables | 7 | Clearly define all outcomes, exposures, predictors, potential confounders, and effect modifiers. Give diagnostic criteria, if applicable  **Key definitions provided in a separate box –page 15-16, with additional explanation in Methods sections (especially section on data collection – page 5).** |
| Data sources/ measurement | 8* | For each variable of interest, give sources of data and details of methods of assessment (measurement). Describe comparability of assessment methods if there is more than one group  **Covered in section on data collection (page 5).** |
| Bias | 9 | Describe any efforts to address potential sources of bias  **Addressed in section “Picaridin repellent, distribution & promotion” and**  **“data collection” (pages 4,5).** |
| Study size | 10 | Explain how the study size was arrived at  **This study reports on one arm of a cluster-randomized trial; we refer to the trial paper and other papers on the project for more information on sample size calculation.** |
| Quantitative variables | 11 | Explain how quantitative variables were handled in the analyses. If applicable, describe which groupings were chosen and why  **Covered in data analysis section in Methods (page 6)** |
| Statistical methods | 12 | (*a*) Describe all statistical methods, including those used to control for confounding  **Covered in data analysis section in Methods (page 6)** |
|  |  | (*b*) Describe any methods used to examine subgroups and interactions  **Not applicable** |
|  |  | (*c*) Explain how missing data were addressed  **There were very few missing data; the analysis is mainly descriptive and it is clarified in the table if information is missing.** |
|  |  | (*d*) If applicable, explain how loss to follow-up was addressed  **Not applicable** |
|  |  | (*e*) Describe any sensitivity analyses  **As the analysis was mainly descriptive, no sensitivity analyses were done.** |
| Results | | |
| Participants | 13* | (a) Report numbers of individuals at each stage of study—eg numbers potentially eligible, examined for eligibility, confirmed eligible, included in the study, completing follow-up, and analysed  **CONSORT flow chart of the cluster randomized trial included** |
|  |  | (b) Give reasons for non-participation at each stage  **CONSORT flow chart of the cluster randomized trial included** |
|  |  | (c) Consider use of a flow diagram  **CONSORT flow chart of the cluster randomized trial included** |
| Descriptive data | 14* | (a) Give characteristics of study participants (eg demographic, clinical, social) and information on exposures and potential confounders  **Study population description: page 8, 18, 20,22** |
|  |  | (*c*) Explain how missing data were addressed  **There were very few missing data; the analysis is mainly descriptive and it is clarified in the tables if information is missing.** |
|  |  | (c) Summarise follow-up time (eg, average and total amount)  **Provided in the tables (see Table 5 on repellent use).** |
| Outcome data | 15* | Report numbers of outcome events or summary measures over time  **Reported in the tables (page 18-22).** |
| Main results | 16 | (*a*) Give unadjusted estimates and, if applicable, confounder-adjusted estimates and their precision (eg, 95% confidence interval). Make clear which confounders were adjusted for and why they were included  **As analysis was essentially descriptive, no confounders were included in analysis.** |
|  |  | (*b*) Report category boundaries when continuous variables were categorized  **Provided in the tables (eg Table 3)** |
|  |  | (*c*) If relevant, consider translating estimates of relative risk into absolute risk for a meaningful time period  **Not applicable** |
| Other analyses | 17 | Report other analyses done—eg analyses of subgroups and interactions, and sensitivity analyses  **All analyses that were conducted are reported in the manuscript.** |
| Discussion | | |
| Key results | 18 | Summarise key results with reference to study objectives  **The discussion starts with the key findings, relating to stated objectives (page 10).** |
| Limitations | 19 | Discuss limitations of the study, taking into account sources of potential bias or imprecision. Discuss both direction and magnitude of any potential bias  **There is a separate section on limitations at the end of the discussion (page 11).** |
| Interpretation | 20 | Give a cautious overall interpretation of results considering objectives, limitations, multiplicity of analyses, results from similar studies, and other relevant evidence  **There are four sections on this in the discussion (page 10 & 11).** |
| Generalisability | 21 | Discuss the generalisability (external validity) of the study results  **This is covered in the section on study limitations (page 11).** |
| Other information | | |
| Funding | 22 | Give the source of funding and the role of the funders for the present study and, if applicable, for the original study on which the present article is based  **Funding statement included (page 12)** |

*Give information separately for exposed and unexposed groups.

**Note:** An Explanation and Elaboration article discusses each checklist item and gives methodological background and published examples of transparent reporting. The STROBE checklist is best used in conjunction with this article (freely available on the Web sites of PLoS Medicine at http://www.plosmedicine.org/, Annals of Internal Medicine at http://www.annals.org/, and Epidemiology at http://www.epidem.com/). Information on the STROBE Initiative is available at http://www.strobe-statement.org.
