# Supplementary material for: Safety of a topical insect repellent (picaridin) during community mass use for malaria control in rural Cambodia
Source: PLoS One. 2017 Mar 24;12(3):e0172566. doi: 10.1371/journal.pone.0172566 (PMC5365103; doi:10.1371/journal.pone.0172566)
Supplement: S4 Text — (PDF) [file pone.0172566.s004.pdf]

Protocol for Reporting, Management and Treatment of Adverse Reactions topical repellents

MalaResT Project (registered ClinicalTrials.gov, number NCT01663831)

1. Contact details for reporting Adverse Reactions

ITM:

Fax: +32 3 247 63 59
E-mail: mcoosemans@itg.be; vsluydts@itg.be; ldurnez@itg.be
Tel: +32 3 247 63 12 or +32 3 247 63 11
Prof. Dr. Marc Coosemans
Dr. Vincent Sluydts
Dr. Lies Durnez
Institute of Tropical Medicine
Nationalestraat 155
B-2000 Antwerp
BELGIUM

CNM:

Principal investigators:
E-mail: sochanthat@cnm.gov.kh; sovannaroths@cnm.gov.kh
Dr. Tho Sochantha (+855 12 881 259)
Dr. Siv Sovannaroth (+855 12 335 029)
CNM
Phnom Penh, Cambodia

DSMB:

Prof.Dr.Lut Lynen
Department of Clinical Sciences, Institute of Tropical Medicine
Nationalestraat 155, B-2000 Antwerp, Belgium
Tel: +32 3 247 64 76
Email: Llynen@itg.be

Immo Kleinschmidt
Department of Infectious Epidemiology, London School of Hygiene and Tropical Medicine, Keppel Street, London WC1E 7HT, UK
Tel: +44 20 79272103
Email: Immo.Kleinschmidt@lshtm.ac.uk

Prof. Antero Aitio
Luoteisväylä 6, 00200 Helsinki, Finland
Tel: +35840 5256285
Email: antero.aitio@fimnet.fi

2. **Contact information of field investigator, his delegate and medical doctor of the referral hospital**

The field investigator is **Dr. Héng Somony (tel +855 12 914 367, somonyps@yahoo.com)**, who is a medical doctor, has a Master of Science degree in Disease Control, and followed a GCP training. He organizes, manages and implements the field activities of the project in close collaboration with CNM, the Provincial staff and Institut Pasteur du Cambodge. He is responsible for supervising the distribution and follow up of the repellents. His delegate for the purpose of this protocol is **Mr. Mean Vanna (+855 92415277 or +855 889984111)** who is a secondary nurse working for the Provincial Health Department of Ratanakiri, for the National Malaria Control Program. The field investigator and his delegate are the key contact persons for reports of adverse events coming from the field. They will perform the field visits (see further).

The medical doctor at the Ratanakiri Referral Hospital is **Mr. Koh Polo (tel +855 12 85 80 85**). He is the key contact person when cases need to be referred to the hospital, and can, when required, also perform field visits.

3. **Scope**

This protocol describes the standardized management of all the suspected adverse reactions (ARs) to the repellent (i.e. picaridin) used in the project 'Repellents as added control measure to long lasting insecticidal nets to target the residual transmission in southeast Asia: a step forwards to malaria elimination'. It describes the way to define, manage, report and follow-up the ARs experienced by all the individuals exposed to the repellent within the study period. The main aim is to ensure the safety of all study participants, as well as the accuracy of the safety reporting in the study.

4. **Picaridin safety information**

The topical repellent that is used in this study is KBR3023 (or Icaridin=Picaridin, brand Autan®). KBR3023 is recommended by 4th WHOPES Working group meeting **for its safety and efficacy** particularly against anopheline mosquitoes [1]. Specifications of the product have been published by WHO in 2004 [2]. The product is registered in several strictly regulated countries, including the USA, Belgium, UK since about 10 years. In Cambodia, the approval by the Cambodian Ethics Committee of the Ministry of Health of the present protocol has provided the clearance for the use of the KBR3023 topical repellent within this study. Two formulations will be used under the brand name of Autan®: a gel formulation of 10% KBR3023 for kids (> 2 years up to 10 years old) and a spray formulation of 20% KBR3023 for adolescents and adults (> 10 years). Table 1 presents the toxicity classification based on animal testing as presented in the npic fact sheet[1].

Table 1: Toxicity classification (based on animal testing) of Picaridin as presented in the npic fact sheet[1]

TOXICITY CLASSIFICATION - PICARIDIN

	High Toxicity	Moderate Toxicity	Low Toxicity	Very Low Toxicity
Acute Oral LD_{50}	Up to and including 50 mg/kg (≤ 50 mg/kg)	Greater than 50 through 500 mg/kg (> 50 – 500 mg/kg)	Greater than 500 through 5000 mg/kg (> 500 – 5000 mg/kg)	Greater than 5000 mg/kg (> 5000 mg/kg)
Inhalation LC_{50}	Up to and including 0.05 mg/L (≤ 0.05 mg/L)	Greater than 0.05 through 0.5 mg/L (>0.05 – 0.5 mg/L)	Greater than 0.5 through 2.0 mg/L (> 0.5 – 2.0 mg/L)	Greater than 2.0 mg/L (> 2.0 mg/L)
Dermal LD_{50}	Up to and including 200 mg/kg (≤ 200 mg/kg)	Greater than 200 through 2000 mg/kg (> 200 - 2000 mg/kg)	Greater than 2000 through 5000 mg/kg (>2000 – 5000 mg/kg)	Greater than 5000 mg/kg (> 5000 mg/kg)
Primary Eye Irritation	Corrosive (irreversible destruction of ocular tissue) or corneal involvement or irritation persisting for more than 21 days	Corneal involvement or other eye irritation clearing in 8 – 21 days	Corneal involvement or other eye irritation clearing in 7 days or less	Minimal effects clearing in less than 24 hours
Primary Skin Irritation	Corrosive (tissue destruction into the dermis and/or scarring)	Severe irritation at 72 hours (severe erythema or edema)	Moderate irritation at 72 hours (moderate erythema)	Mild or slight irritation at 72 hours (no irritation or erythema)

The highlighted boxes relfect the values in the "Acute Toxicity" section of this fact sheet. Modeled after the U.S. Environmental Protection Agency, Office of Pesticide Programs, Label Review Manual, Chapter 7: Precautionary Labeling. http://www.epa.gov/oppfead1/labeling/lrm/chap-07.pdf

When used at normal preventive dosage in humans, two non-serious, treatable adverse reactions have been described in literature:

- Allergic contact dermatitis [3]: A 39-year-old man developed allergic contact dermatitis several hours after using a repellent containing 10% picaridin and methylglucose dioleate. Patch tests suggested that the man had reacted to both the picaridin and the methylglucose dioleate in the product.
- Burning sensation [4]: 42 (11.9%) out of 353 responses in Australian soldiers reported a burning sensation after the use of 19.2% picaridin non-pressurised pump action spray which is about half as much as was reported by DEET users (22.3%). DEET is the most commonly used topical repellent in the world.

Thus, allergic contact dermatitis (which according to an international classification system, CTC[2] version 2.0, could be categorized under mild or moderate pruritus, skin rash, allergic reaction or urticaria), and eye irritation (mild or moderate keratitis) in case the repellent gets into the eye, will be considered as expected adverse reactions (see definitions below) from picaridin use.

[1] npic fact sheet for picaridin available from: http://npic.orst.edu/factsheets/Picaridintech.pdf

[2] CTC version 2.0 available from http://ctep.cancer.gov/protocolDevelopment/electronic_applications/docs/ctcv20_4-30-992.pdf

5. Definitions

This protocol covers the definitions, management, reporting and treatment of suspected **adverse reactions (ARs)**, which are adverse events (AEs) that are possibly, probably or definitely related with to the use of the picaridin within the study protocol (see below).

An **adverse event** (AE) is defined, for the scope of this study, as any untoward, undesired, or unplanned event in the form of signs, symptoms, disease, or laboratory or physiologic

observations occurring in a human being in a temporal relationship to the use of picaridin, regardless of causal relationship, including any clinically important worsening of a pre-existing condition.

Product (picaridin) Related: An adverse event will be considered "product related" if the field investigator or his delegate assesses the adverse event(s) as possibly, probably, or definitely related. In that case it will be defined as a suspected **adverse reaction (AR)**. An adverse event will be considered "not product or related" if the field investigator or his delegate assess the adverse event(s) as "probably not related" or "definitely not related". Whenever this assessment is unknown or unclear, or if the opinions of the field investigator or his delegate are discrepant, the adverse event(s) will be treated as product related.

- **Definitely Related** The event can be fully explained by administration of the picaridin and/or a re-challenge was positive.
- **Probably Related** The event is more likely to be explained by administration of the picaridin rather than the patient/subject's clinical state or other agents/therapies.
- **Possibly Related** The event may be explained by administration of the picaridin, or by the patient/subject's clinical state or other agents/therapies.
- **Probably Not Related** The event is more likely to be explained by the patient/subject's clinical state or other agents/therapies rather than the picaridin.
- **Definitely Not Related** The event can be fully explained by the patient/subject's clinical state or other agents/therapies.

An **expected adverse reaction** is one that is listed in the current product labeling or technical sheet or that is reported in literature. For picaridin, as described in section 4, allergic contact dermatitis as well as eye irritation are considered expected product related adverse reactions.

An **unexpected adverse reaction** (UAR) is one that is not listed in the current product labeling or technical sheet and was not mentioned in section 4 of this document. An UAR may also include any AR that are symptomatically and pathophysiologically related to known reactions, but that differs from the known reactions because of greater severity or specificity. For example, allergic contact dermatitis is an expected AR, as described above. However, anaphylaxis would be an unexpected AR (by virtue of greater severity). For the scope of this project, UARs will be reported and managed in the same way as the expected ARs, except when they fulfill the definition of a serious adverse reaction (see below).

A **serious adverse reaction** (SAR) is defined as an AR which also

- is life threatening
  Life threatening refers to immediate risk of death as the event occurred. A life-threatening experience does not include an experience that, had it occurred in a more severe form, might have caused death but as it actually occurred did not create an immediate risk of death
- requires inpatient hospitalization (i.e. overnight) or prolongation of an existing hospitalization;

- results in a persistent or significant disability or incapacity (substantial disruption in a person's ability to conduct normal life functions);
- results in cancer;
- results in a congenital anomaly or birth defect.

Additionally, important medical reactions that may not be life threatening, or require hospitalization may be considered SARs when, based on appropriate medical judgment, they may jeopardize the patient or subject and may require medical or surgical intervention to prevent one of the outcomes listed in this definition. In this protocol, ARs classified as severity grade 4, i.e. with life-threatening consequences, according to the CTC version 2.0 (see Annex 11.5) are considered SARs.

Important note: SARs are very unlikely to occur considering the safety of picaridin, which have been documented before registration and during the more than 10 years of use in many countries. However, their management is included in this protocol in order to safeguard the safety of the participants at all times.

6. Information flow, visit decisions, and actions taken

An AR must be recorded from the time that the subject begins to use picaridin for the scope of the study to one month after completion of the final survey in October/November of 2012 or 2013 (the last month, by passive reporting through the distributors). All ARs will be recorded on the source documents (see below). This AR information will be collected **on a regular basis** during the study, as described in the study protocol. A report of the ARs is prepared at least every three months, except for the SARs that should be reported immediately (see below). The field investigator and the principal investigators at CNM are responsible to ensure and verify that each of these events is managed appropriately, and followed up until resolution.

The actions to be taken are described in Annex 11.3, and the recommended treatment of the expected ARs is described in Annex 11.5. Case management costs will be covered by the project budget and, if needed, by its insurance.

The information flow and visit decisions are schematically shown in Annex 11.4 and summarized below.

The distributors in the villages, as specified in the study protocol, are the key contact persons for the people wanting to report perceived adverse reactions related to the repellent use and they have been trained specifically for this task. The distributors should report them to the field investigator or his delegate (see their details in section 2), and they should take actions in the following way (according to the severity):

6.1. ARs other than SARs reported by phone to the field investigator

Distributors are trained to contact the field investigator immediately by phone in case study subjects report ARs perceived to be related to picaridin use in the period between two consecutive picaridin distributions. In this case, the field investigator or his delegate will talk to the patient or the distributor by phone to assess the severity of the case, advice the user to stop the use of the repellent, schedule a visit if treatment or refer to the health center or hospital if considered necessary (according to Annex 11.3, and 11.4). In case a visit is required, the investigation reports (Form B) should be filled in and treatment will be given if necessary (according to Annex 11.5).

6.2. ARs reported in the household distribution sheets in the bi-weekly meetings

At each distribution, as mentioned in the study protocol all ARs perceived by the study subjects and that occurred in the previous two weeks are recorded by the distributor in the reported side effect column of the **household distribution sheet (HDS)** (FORM: 'Distribution of repellent bottles and recovery of empty bottles' in Annex 6 of the study protocol part 2). The information recorded is based on the narrative of the household representative and is noted in key words. All HDSs are brought to the health centers (HC) by the distributors during the bi-weekly meeting with the distributor supervisors (DS). The field investigator and his delegate take turn to visit different HCs during the meetings to review HDSs and investigate all perceived ARs. In addition, the field project team schedules the visits to the villages that never reported ARs and the villages from which the distributors are absent from the bi-weekly meeting.
Based on the information given in the HDSs or, if necessary, based on additional information provided by the patient by phone, a visit to the patient by the field investigator or his delegate will be scheduled, referral to the health center or hospital will be advised or advice will be given to stop using the repellent (Annex 11.3, 11.4), as described in 6.1. In case a visit is required, the investigation reports (Form B) should be filled in and treatment will be given if necessary (according to Annex 11.5).

6.3. SARs & ARs to be reported immediately

SARs are to be reported immediately (see below). ARs that must be reported in the same time frame and following the same reporting process as SARs are:

- All suspected **ARs occurring in pregnant women**. These reports should be followed up for information about the course of the pregnancy and delivery, as well as the condition of the newborn. When the newborn is healthy, additional follow-up is not needed. This follow-up information should be provided to the CNM, ITM, and DSMB following the procedures for SARs as described below.
- Picaridin **abuse and overdose** with or without ARs. It is up to the participating investigator to decide whether a dose was or might be an overdose.
- ARs occurring **after unauthorized use** (e.g. by people given the medical advice to stop using picaridin) and accidental use in persons not participating in the study (i.e. under 2 year olds).

Distributors are trained to inform the field investigator immediately by phone if any SARs or other ARs that should be reported immediately occur. In case of any doubt the distributor will take all precautions and inform the field investigator immediately by phone.

In case of life threatening symptoms suspected, the patients must be immediately and directly referred to the Ratanakiri Referral Hospital (Director Hing Phan Sakunthea tel +855 11 52 80 08). The contact person is the chief of technical office, **Mr. Koh Polo,** medical doctor (tel **+855 12 85 80 85**). This hospital has the facilities to deal with the expected adverse reactions (including unexpected cases of anaphylactic shock). The hospital has three well equipped ambulances to transfer the patients from the village to the Ratanakiri Referral Hospital, and, if necessary, to Yalay Provincial hospital in Vietnam. Case management costs related to adverse reactions, will be in charge of the project. The subject will be observed and monitored carefully until the condition resolves or stabilizes.

In other cases, the patients must be urgently and directly be referred to the nearest health center or investigated by the field investigator, his delegate, or the medical doctor from the Ratanakiri Referral Hospital. Actions for immediate treatment and relief should be undertaken and documented (Annex 11.3, form B).

Any SAR or other immediately reportable ARs, as well as any follow-up information, must be reported to the principal investigators at CNM and to the ITM focal persons (**no later than 24 working hours** after the investigator becomes aware of the SAR) by faxing or e-mailing a completed serious adverse reaction form (Form A in Annex 11.1) to the numbers or e-mail addressed indicated in the front of this protocol. The ITM focal persons will forward this report to the three members of the DSMB (coordinates mentioned under section 1). Telephone confirmation that the form was received should be sought by those who do not acknowledge receipt by e-mail.

The CNM principal investigators are responsible to inform the Ethical Committee of Cambodia by means of aggregated data on a yearly basis. In the same way, the ITM focal persons are responsible to inform the ITM Institutional Review Board and the Ethics Committee of the Antwerp University Teaching Hospitals by means of aggregated data on a yearly basis. They are also responsible to ensure appropriate follow-up by the DSMB, and to ensure timely and complete communication among all the concerned counterparts: the field, the CNM principal investigators, the ECs, the DSMB and, when appropriate, the manufacturer of picaridin. The manufacturer may be requested to advice on the causality assessment of specific suspected AR, based on the information they have in their safety database.

7. Recording in the Form B

For all ARs visited by the field investigator, or his assisting medical staff, the information is recorded in Form B (Annex 11.2) by the visiting person. The information recorded should be based on the signs and symptoms detected during the physical examination and clinical evaluation of the subject. In addition to the information obtained from those sources, the subject should be asked the following nonspecific question: “How have you been feeling

since you use the repellent?". Signs and symptoms should be recorded using standard medical terminology.

The **following AE information** must be included (as soon as available):

- Diagnosis
    - The specific condition or event
    - The evolution of the condition (according to the investigator or patient: improving, worsening or status quo);
- Whether the condition was pre-existing (acute or chronic) before the first use of the picaridin, and if so, whether it has worsened (e.g. in severity and/or frequency);
- The dates and times of occurrence (if not accurate, the approximate duration may be indicated):
    - Date of event onset
    - Date event resolved
    - If applicable, also mention the date of interruption of use of picaridin and/or the date of restart
- Severity of the event according to CTC[1] version 2.0 grading scale (listed in Annex 11.5 for the expected ARs)
    - Grade 1: Mild
    - Grade 2: Moderate
    - Grade 3: Severe
    - Grade 4: Life-threatening consequences
- Causal relationship to picaridin use: This will be determined by the reporting investigator on the basis of his or her clinical judgment and the definitions mentioned in 5. When assessing the relationship between the repellent use and an AE, the following should be considered:
    - Temporal relationship between administration of the picaridin and the AE
    - Biological plausibility of relationship
    - Subject's underlying clinical state or concomitant agents and/or therapies
    - When applicable, whether the AE abates on discontinuation of picaridin (dechallenge)
    - When applicable, whether the AE reappears on repeat exposure to picaridin (rechallenge).
- Action taken
    - Treatment: yes/no. If yes, define which treatment (according to Annex 11.5)
    - Advice to stop using picaridin or not
- Outcome : assessed according to the following classification:
    - Resolved, No sequel: The patient has fully recovered with no observable residual effects
    - AR still present-no treatment: Improvement in the patient's condition has occurred, but the AR does not need treatment
    - AR still present-being treated: Improvement in the patient's condition has occurred, but the AR is still under treatment

- Residual effects present-not treated: The residual effects, although present, do not need treatment
- Residual effects present-treated: The residual effects are still present and require treatment
- Deterioration: The patient's overall condition has worsened
- Permanent damage: The AR has resulted in a permanent impairment
- Death: The patient died (any reason)
- Unknown: The outcome of the AR is not known because the patient is lost to follow-up

[1]CTC version 2.0 available from http://ctep.cancer.gov/protocolDevelopment/electronic_applications/docs/ctcv20_4-30-992.pdf

ANNEX 11.1: FORM A-SERIOUS ADVERSE REACTION REPORT FORM

Please complete the information requested and forward to below addresses not later than 24 working hours after becoming aware of the serious Adverse Reaction (and confirm by telephone that the form is received by those recipients who do not acknowledge receipt):

ITM:

Fax: +32 3 247 63 59
E-mail: mcoosemans@itg.be; vsluydts@itg.be; ldurnez@itg.be
Tel: +32 3 247 63 12 or +32 3 247 63 11
Prof. Dr. Marc Coosemans
Dr. Vincent Sluydts
Dr. Lies Durnez
Institute of Tropical Medicine
Nationalestraat 155
B-2000 Antwerp
BELGIUM

CNM:

Principal investigators:
E-mail: sochanthat@cnm.gov.kh; sovannaroths@cnm.gov.kh
Dr. Tho Sochantha (+855 12 881 259)
Dr. Siv Sovannaroth (+855 12 335 029)
CNM
Phnom Penh, Cambodia

DSMB:

Prof.Dr.Lut Lynen
Department of Clinical Sciences, Prince Leopold Institute of Tropical Medicine
Nationalestraat 155, B-2000 Antwerp, Belgium
Tel: +32 3 247 64 76
Email: Llynen@itg.be

Immo Kleinschmidt
Department of Infectious Epidemiology, London School of Hygiene and Tropical Medicine, Keppel Street, London WC1E 7HT, UK
Tel: +44 20 79272103
Email: Immo.Kleinschmidt@lshtm.ac.uk

Prof. Antero Aitio
Luoteisväylä 6, 00200 Helsinki, Finland
Tel: +35840 5256285
Email: antero.aitio@fimnet.fi

PROTOCOL TITLE: Repellents as added control measure to long lasting insecticidal nets to target the residual transmission in southeast Asia: a step forwards to malaria elimination	
PRINCIPAL INVESTIGATOR:<br><br>***Prof. Dr. Marc Coosemans***<br><br>***Dr. Tho Sochantha***<br><br>***Dr. Siv Sovannaroth***	Institute: of Tropical Medicine Office Phone: +32 3 247 63 12 or +32 3 247 63 11 Fax:+32 3 247 63 59<br>E-mail: mcoosemans@itg.be; ldurnez@itg.be; vsluydts@itg.be<br><br>CNM Phone: +855 12 881 259: +855 12 335 029<br>E-mail: sochanthat@cnm.gov.kh; sovannaroths@cnm.gov.kh

<table>
<tr><td>DATE OF VISIT:</td><td colspan="2">____/____/_____</td></tr>
<tr><td>LOCATION OF VISIT:</td><td colspan="2"></td></tr>
<tr><td>BRIEF DESCRIPTION OF SUBJECT(S)
(Do NOT include identifiers.)</td><td colspan="2">SEX: AGE: ___________
Diagnosis:</td></tr>
<tr><td colspan="3">DESCRIPTION OF THE NATURE OF THE SERIOUS ADVERSE REACTION AND THE EVOLUTION OF THE CONDITION</td></tr>
<tr><td>CATEGORY (outcome) OF THE SERIOUS ADVERSE REACTION:
[ ] death
[ ] disability/incapacity
[ ] life-threatening
[ ] congenital anomaly/birth defect
[ ] hospitalization-initial or prolonged
[ ] required intervention to prevent permanent impairment
[ ] other</td><td colspan="2">RELATIONSHIP OF SERIOUS ADVERSE REACTION TO RESEARCH:
[ ] 1 = definitely related
[ ] 2 = probably related
[ ] 3 = possibly related</td></tr>
<tr><td colspan="2">INVESTIGATOR'S SIGNATURE:</td><td>DATE:</td></tr>
</table>

ANNEX 11.2: FORM B-ADVERSE EVENT REPORT FORM

PROTOCOL TITLE: Repellents as added control measure to long lasting insecticidal nets to target the residual transmission in southeast Asia: a step forwards to malaria elimination

Date: / / (dd/mm/yyyy)		Name of visiting medical doctor:
Individual identification number:	Village:	Adverse Event case number (Village Code/unique number):

Case Description (Answer to the question 'How has the individual been feeling since he/she used the repellent'). If possible, illustrate with a picture.

Did the individual patient receive treatment? ☐Yes ☐ No

If yes, what treatment:

Did the patient stop using the repellent? ☐Yes ☐ No If yes, approximate date of stop using (DD/MM/YYYY): / /

Did the patient use the repellent again after? ☐Yes ☐ No If yes, approximate date of retry (DD/MM/YYYY):): / /

If yes, did the symptoms reappear? ☐Yes ☐ No

**Severity (according to CTCAE grading)**	**Improving**	**Pre-existing**	**Study Intervention Relationship**	**Action Taken Regarding Study Intervention**	**Outcome of AE**	**Expected**	**Serious**
1 = Grade 1<br>2 = Grade 2<br>3 = Grade 3<br>4 = Grade 4	1 = Yes<br>2 = No<br>3 = unclear	1 = Yes, but did not get worse<br>2 = Yes, did get worse<br>3 = No<br>4 = unclear	1 = definitely related<br>2 = probably related<br>3 = possibly related<br>4 = probably not related<br>5 = definitely not related	1 = None<br>2 = Discontinued repellent use permanently<br>3 = Discontinued repellent use temporarily	1 = Resolved, No Sequel<br>2 = AE still present- no treatment<br>3 = AE still present-being treated<br>4 = Residual effects present-not treated<br>5 = Residual effects present- treated<br>6 = Deterioration<br>7 = Permanent damage<br>8 = Death<br>9 = Unknown	1 = Yes<br>2 = No	1 = Yes (Grade 4)<br>2 = No (Grade 1, 2 or 3)<br>(If yes, complete SAE form)

Adverse Event (Diagnosis)	Start Date (or approximate period)	Stop Date (or approximate period)	Severity	Improving?	Pre-existing?	Relationship to Study Treatment	Action Taken	Outcome of AE	Expected?	Serious Adverse Event?	Initials
1.											
2.											
3.											

ANNEX 11.3: GENERAL ACTIONS TO BE TAKEN FOR ARs

Adverse Reactions

Mild (Grade 1)

1. **Rinse skin with water**
2. **Avoidance:** Discontinue the repellent use permanently in case of definitely related. In case of possibly or probably repellent related, reassess reuse after symptoms completely disappear if agreed by patient. If symptoms reappear, stop permanently.
3. **If no spontaneous recovery, active treatment indicated**

Moderate (Grade 2)

1. **Rinse skin with water**
2. **Active treatment indicated**
3. **Avoidance:** Discontinue the repellent use permanently in case of definitely related. In case of possibly or probably repellent related, reassess reuse after symptoms completely disappear if agreed by patient. If symptoms reappear, stop permanently.

Field investigator, his delegate, or health center

Severe (Grade 3)

1. **Rinse skin with water**
2. **Refer the patient to the Ratanakiri Provincial Hospital for active treatment**
3. **Avoidance:** Discontinue the repellent use permanently

Life-treathening consequences (Grade 4)

REFER THE PATIENT IMMEDIATELY TO THE RATANIKIRI PROVINCIAL HOSPITAL[1]

Avoidance: Discontinue the repellent use permanently

Field investigator, his delegate, or health center

[1] Referral is done to the Ratanakiri Referral hospital (Director Hing Phan Sakunthea tel 011 52 80 08). The contact person is the chief of technical office, **Mr. Koh Polo,** medical doctor (tel **012 85 80 85**). This hospital has the facilities to deal with the expected adverse reactions (including unexpected cases of anaphylactic shock). The

hospital has three well equipped ambulances to transfer the patients from the village to the Ratanakiri Referral hospital, and, if necessary, to Yalay Provincial hospital in Vietnam. Case management costs related to adverse reactions, will be in charge of the project (insurance of ITM).

ANNEX 11.4: VISIT DECISION TREE

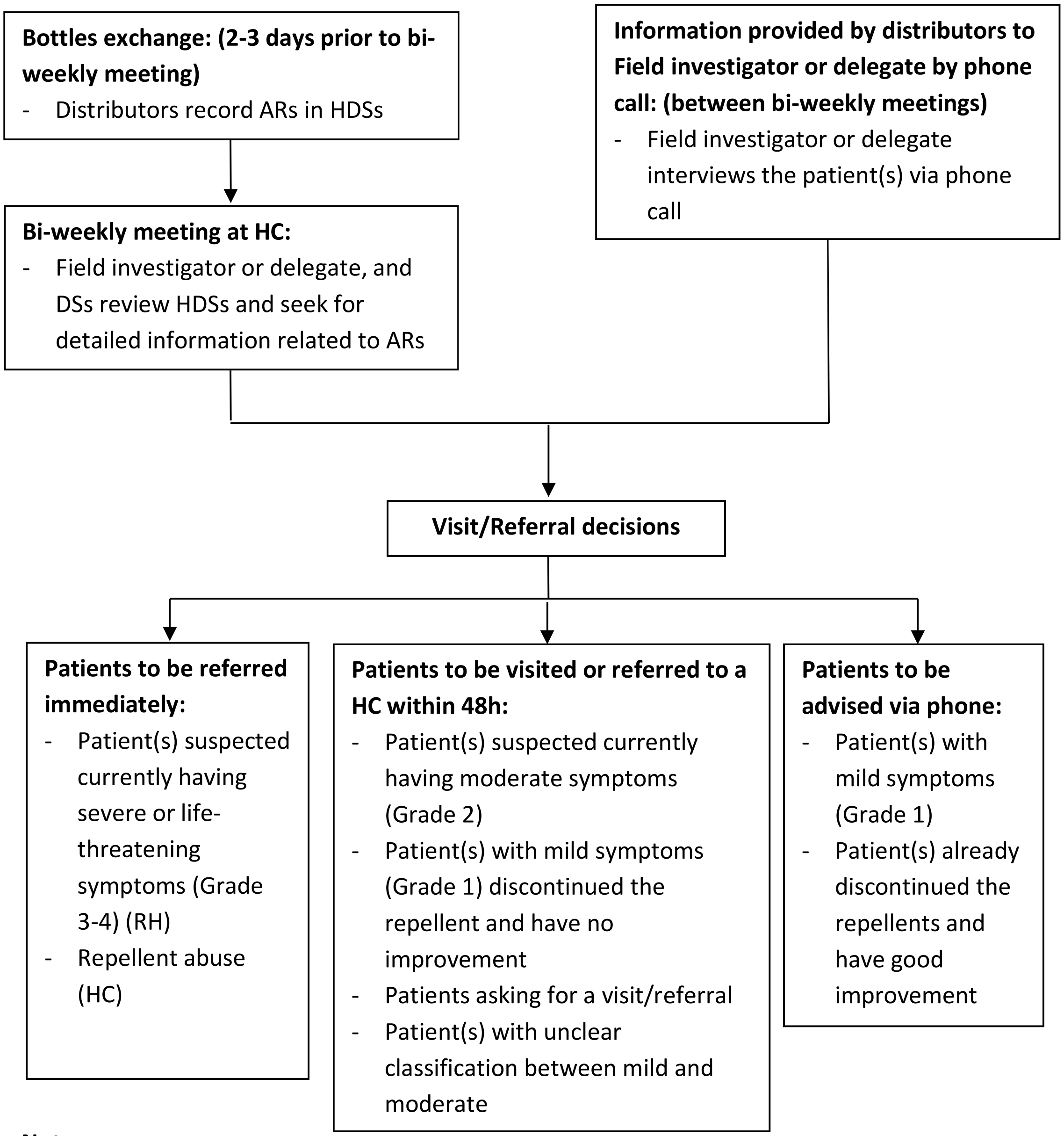

Note:

AR: suspected adverse reaction
HC: health center
DS: Distributor Supervisor
HDS: Household Distribution Sheet
RH: Ratanakiri Referral hospital

ANNEX 11.5: GRADING SCALE OF EXPECTED ADVERSE REACTIONS ACCORDING TO THE CTC[1] Version 2.0 AND PROPOSED TREATMENT

Important note: Only Grade 1 & 2 are expected ARs. Grade 3 & 4 are not observed previously and are mentioned here for completeness

Adverse Event		Grade 1	Grade 2	Grade 3	Grade 4
Pruritus (itching)	**Symptoms:**	mild or localized, relieved spontaneously or by local measures	intense or widespread, relieved spontaneously or by systemic measures	intense or widespread and poorly controlled despite treatment	-
	**Treatment:**	▪ Local measurement: rinse skin with water, wet cloth<br>▪ If no improvement (>6 hours):<br>• Anti-histamine e.g. Chlorpheniramine[2]	▪ Local measurement: rinse skin with water, wet cloth<br>▪ Anti-histamine e.g. Chlorpheniramine[2]	▪ Refer to the hospital	-
Rash/desquamation<br><br>Note: Also consider Allergic reaction/hypersensitivity.	**Symptoms:**	macular or papular eruption or erythema without associated symptoms	macular or papular eruption or erythema with pruritus or other associated symptoms covering <50% of body surface or localized desquamation or other lesions covering <50% of body surface area	symptomatic generalized erythroderma or macular, papular or vesicular eruption or desquamation covering ≥50% of body surface area	generalized exfoliative dermatitis or ulcerative dermatitis
	**Treatment:**	▪ Local measurement: rinse skin with water, wet cloth<br>▪ Topical corticosteroid e.g. Triamcinolone [2]	▪ Local measurement: rinse skin with water, wet cloth<br>▪ Topical corticosteroid e.g. Triamcinolone[2]	▪ Refer to the hospital	▪ Refer to the hospital
Allergic reaction/hypersensitivity (including drug fever)	**Symptoms:**	transient rash, drug fever <38°C (<100.4°F)	urticaria, drug fever ≥38°C (≥100.4°F), and/or asymptomatic bronchospasm	symptomatic bronchospasm, requiring parenteral medication(s), with or without urticarial; allergy-related edema/angioedema	anaphylaxis
Note: Isolated urticaria, in the absence of other manifestations of an allergic or hypersensitivity reaction, is graded	**Treatment:**	▪ Local measurement: rinse skin with water, wet cloth<br>▪ Anti-histamine e.g. Chlorpheniramine[2]	▪ Local measurement: rinse skin with water, wet cloth<br>▪ Anti-histamine e.g. Hydroxyzine[2] & oral corticosteroid e.g. prednisolone[2]	▪ Refer to the hospital	▪ Refer to the hospital
Urticaria (hives, welts, wheals)	**Treatment:**	requiring no medication	requiring PO or topical treatment or IV medication or steroids for <24 hours	requiring IV medication or steroids for ≥24 hours	-
Keratitis (corneal inflammation/ corneal ulceration)	**Symptoms:**	abnormal ophthalmologic changes but asymptomatic or symptomatic without visual impairment (i.e., pain and irritation)	symptomatic and interfering with function, but not interfering with activities of daily living	symptomatic and interfering with activities of daily living	unilateral or bilateral loss of vision (blindness)
	**Treatment:**	▪ Rinse eye with saline water	▪ Rinse eye with saline water<br>▪ Anti-histamine e.g. Chlorpheniramine[2]<br>▪ If not better refer the patient to an ophthalmologist or hospital	▪ Refer to the hospital	▪ Refer to the hospital

[1] CTC version 2.0 available from http://ctep.cancer.gov/protocolDevelopment/electronic_applications/docs/ctcv20_4-30-992.pdf

[2] Dosage according to the specific product labeling

References

1. WHOPES: **Report of the 4th WOPES Working Group meeting: IR3535, KBR3023, (RS)-methoprene 20% EC, pyriproxifen 0.5% GR and lambda-cyhalothrin 2.5% CS. 4-5 December 2000**. In. Edited by Organization WH, vol. WHO/CDS/WHOPES/2001.2. Geneva; 2001.
2. WHO: **Specifications and evaluations for public health pesticides. Icaridin**. 2004, <http://www.who.int/whopes/quality/en/Icaridin_spec_eval_Oct_2004.pdf>.
3. Corazza M, Borghi A, Zampino MR, Virgili A: **Allergic contact dermatitis due to an insect repellent: double sensitization to picaridin and methyl glucose dioleate**. *Acta Derm Venereol* 2005, **85**(3):264-265.
4. Frances SP, Cooper RD: **Personal protection measures against mosquitoes**. *ADF Health* 2002, **3**:58-63.
