# Supplementary material for: Safety of a topical insect repellent (picaridin) during community mass use for malaria control in rural Cambodia
Source: PLoS One. 2017 Mar 24;12(3):e0172566. doi: 10.1371/journal.pone.0172566 (PMC5365103; doi:10.1371/journal.pone.0172566)
Supplement: S1 Table — (DOCX) [file pone.0172566.s005.docx]

**Distribution of repellent bottles and recovery of empty bottles**

Village: ___________ Commune:___________ Family code: |___|___|___|___|/|___|___|___|___|

Signature/finger print of family representative Signature/finger print of distributor
