# Supplementary material for: Safety of a topical insect repellent (picaridin) during community mass use for malaria control in rural Cambodia
Source: PLoS One. 2017 Mar 24;12(3):e0172566. doi: 10.1371/journal.pone.0172566 (PMC5365103; doi:10.1371/journal.pone.0172566)
Supplement: S2 Table — (DOCX) [file pone.0172566.s006.docx]

**Summary of adverse reactions and reactions probably not related to the repellent use individually reported**

| **No** | **Sex** | **Age** | **Picaridin concentration** | **Administration route** | **Reaction time** | **Symptoms** | **Severity** | **Picaridin relationship** | **Expected adverse reaction*** |
| --- | --- | --- | --- | --- | --- | --- | --- | --- | --- |
| ***Adverse reactions*** | | | | | | | | | |
| 01. | M | 17 | 20% | Cutaneous | Not precise (in the same morning) | Itchy and painful pustules | Mild | Definitely related | Yes |
| 02. | M | 2 | 10% | Cutaneous | Immediately | Itchy without rash | Mild | Definitely related | Yes |
| 03. | F | 60 | 20% | Cutaneous | Few hours | Itchy rash, papules, pustules | Mild | Definitely related | Yes |
| 04. | F | 60 | 20% | Cutaneous | 5 days of continuous use | Itchy rash | Mild | Definitely related | Yes |
| 05. | F | 60 | 20% | Cutaneous | Not precise | Itchy rash, discrete eczematous rash | Mild | Definitely related | Yes |
| 06. | F | NA | 20% | Cutaneous | Immediately | Itchy without rash | Mild | Definitely related | Yes |
| 07. | F | NA | 20% | Cutaneous | Immediately | Itchy without rash | Mild | Definitely related | Yes |
| 08. | F | NA | 20% | Cutaneous | Immediately | Itchy without rash | Mild | Definitely related | Yes |
| 09. | M | 40 | 20% | Cutaneous | Immediately | Itchy and painful vesicular rash | Mild | Definitely related | Yes |
| 10. | M | 26 | 20% | Cutaneous | Immediately | Skin irritation without rash | Mild | Definitely related | Yes |
| 11. | F | 30 | 20% | Cutaneous | Immediately | Skin irritation, itchy | Mild | Definitely related | Yes |
| 12. | F | 33 | 20% | Cutaneous | 6 minutes | Itchy rash | Mild | Definitely related | Yes |
| 13. | F | 29 | 20% | Cutaneous | 10 minutes | Itchy rash | Mild | Definitely related | Yes |
| 14. | M | 60 | 20% | Cutaneous | Immediately | Feeling bitter taste | Mild | Possibly related | No |
| 15. | F | 37 | 20% | Cutaneous | Immediately | Skin irritation, feet oedema, itchy rash, flaky skin | Mild | Definitely related | Yes |
| 16. | F | 29 | 20% | Cutaneous | Immediately | Skin irritation, itchy pustules | Mild | Definitely related | Yes |
| 17. | F | 16 | 20% | Cutaneous | Immediately | Skin irritation, itchy macules and papules | Mild | Definitely related | Yes |
| 18. | M | 3 | 10% | Cutaneous | Immediately | Itchy papules and pustules | Mild | Definitely related | Yes |
| 19. | F | 22 | 20% | Cutaneous | Immediately | Skin irritation, burn-like blisters, scars of papules | Mild | Definitely related | Yes |
| 20. | M | 23 | 20% | Cutaneous | 3 days of continuous use | Itchy rash, painful papules, scars of macule and papules | Mild | Definitely related | Yes |
| 21. | F | 10 | 10% | Cutaneous | Immediately | Itchy papules and pustules (preexisting scabies) | Mild | Definitely related | Yes |
| 22. | M | 78 | 20% | Cutaneous | 2 hours after profuse use | Itchy, swollen (scratched) hand | Mild | Probably related | Yes |
| ***Reactions probably not related to the repellent use*** | | | | | | | | | |
| 23. | F | 31 | 20% | Cutaneous | About 10 hours | Sticky feeling on the tongue (R) | Mild | Probably not related | No |
| 24. | M | 40 | 20% | Cutaneous | Immediately | Shortness of breath (R) | Mild | Probably not related | No |
| 25. | M | 4 | 10% | Cutaneous | 15 minutes | Red itchy rash (R) | Mild | Probably not related | Yes |
| 26. | F | 29 | 20% | Cutaneous | Not precise | Red dot (R) | Mild | Probably not related | Yes |
| 27. | M | 40 | 20% | Cutaneous | Not precise | Itchy rash (R) | Mild | Probably not related | Yes |
| 28. | F | 29 | 20% | Cutaneous | Not precise | Itchy rash (R) | Mild | Probably not related | Yes |
| 29. | F | 31 | 20% | Cutaneous | Not precise | Itchy rash (R) | Mild | Probably not related | Yes |
| 30. | F | 3 | 10% | Cutaneous | More than 10 hours | Vomiting (R) | Mild | Probably not related | No |

*Note:* (R): reported by the patients but not confirmed during the visit of medical doctors.

* Expected adverse reaction: mild to moderate allergic contact dermatitis and eye irritation. Unexpected adverse reaction: any other mild to moderate reactions as mentioned above and any severe adverse reactions.
