# Supplementary material for: Safety of a topical insect repellent (picaridin) during community mass use for malaria control in rural Cambodia
Source: PLoS One. 2017 Mar 24;12(3):e0172566. doi: 10.1371/journal.pone.0172566 (PMC5365103; doi:10.1371/journal.pone.0172566)
Supplement: S3 Table — (DOCX) [file pone.0172566.s007.docx]

**Summary of cases of repellent abuse (oral ingestion)**

| **No.** | **Sex** | **Age** | **Picaridin concentration** | **Administration route** | **Quantity taken** | **Reaction time** | **Symptoms** | **Severity** | **Hospitalized** | **Type of use** |
| --- | --- | --- | --- | --- | --- | --- | --- | --- | --- | --- |
| 01. | F | 26 | 20% | Oral | 100 ml | Immediately | Vomiting, weakness | Mild | No | Suicide |
| 02. | M | 7 | 20% | Oral | A mouth full | 30 minutes | Headache, buzzing in ears, abdominal pain, abdominal distension, dizziness, sore throat | Moderate | No | Accident |
| 03. | M | 5 | 20% | Oral | A mouth full | 30 minutes | Headache, buzzing in ears, abdominal pain, abdominal distension, dizziness, sore throat | Moderate | No | Accident |
| 04. | M | 4 | 20% | Oral | A mouth full | Immediately | Vomiting | Mild | No | Accident |
| 05. | M | 61 | 20% | Oral | A mouth full | No reaction | None | no reaction | No | Accident |
| 06. | F | 25 | 20% | Oral | 50 ml | Immediately | Headache, weakness (4-months preganant) | Mild | No | Suicide |
| 07. | F | 19 | 20% | Oral | 300 ml | Immediately | Vomiting, hallucination (2 hrs.) | Life-threatening | Yes | Suicide |
| 08. | F | 25 | 20% | Oral | 50 ml | Immediately | Nausea, headache, dizziness, weakness | Moderate | Yes | Suicide |
| 09. | F | 13 | 20% | Oral | 200 ml | Immediately | Headache, sore throat, vomiting, dazzled vision, dizziness, abdominal pain, chest pain, difficulty breathing, unconsciousness (30 min) | Life-threatening | Yes (private) | Suicide |
| 10. | M | 2 | 20% | Oral | A mouth full | 1-2 hours | Nausea, hyper-salivation, oedema of lips | Mild | No | Accident |
| 11. | M | 29 | 20% | Oral | A mouth full (not swallowed) | Immediately | Vomiting | Mild | No | Accident |

*Note:* All cases were due to oral ingestion, definitely related to picaridin use, unexpected adverse reaction (see definition in Box 1), and completely recovered.
